# Choroidal changes and associations with visual acuity in diabetic patients

**DOI:** 10.1186/s40942-021-00355-z

**Published:** 2022-01-08

**Authors:** João Heitor Marques, Ana Marta, Catarina Castro, Pedro Manuel Baptista, Diana José, Daniel Almeida, António Ribeiro, Irene Barbosa

**Affiliations:** 1 Ophthalmology Department, Centro Hospitalar Universitário do Porto, Largo Prof. Abel Salazar, 4099-001 Porto, Portugal; 2 Instituto de Ciências Biomédicas Abel Salazar, Universidade do Porto, Porto, Portugal

**Keywords:** Choroid, Choroidal thickness, Choroidal vascularity index, Diabetes, Diabetic retinopathy

## Abstract

**Background:**

The variable visual function observed in diabetic retinopathy (DR) patients is not fully explained by the classic staging system. Our purpose was to evaluate choroidal changes, in standardized sectors, in DR patients and to find associations between choroidal measurements and visual function.

**Methods:**

Cross-sectional study that included the right eye of diabetic patients (n = 265) without active edema, ischemia or neovascularization and age-matched controls (n = 73). Optical coherence tomography (OCT) imaging was performed with enhanced depth imaging protocol. Choroidal vascularity index (CVI) was calculated in a 5 mm scan centered in the fovea.

**Results:**

CVI decreased with age (p < 0.001) but was not influenced by axial length. A multivariate analysis adjusting for age confirmed a significant difference in CVI between DR eyes that had previous treatments (intravitreal injections and/or photocoagulation) compared to control eyes (p = 0.013) and to DR eyes that never required treatment (p = 0.002). There was no significant difference between non-DR diabetic patients and normal controls. Considering the group of DR patients that had previous treatments, in eyes without optic media opacification, BCVA correlated with CVI (r = − 0.362, p < 0.001), whereas full retina thickness and individual retinal layer thickness did not (p > 0.066).

**Conclusions:**

A reduction in CVI was observed in patients with a more advanced stage of DR. In treated DR patients with stable disease, choroidal biomarkers correlated with best-corrected visual acuity whereas retinal biomarkers did not.

*Trial registration*: N/A

**Supplementary Information:**

The online version contains supplementary material available at 10.1186/s40942-021-00355-z.

## Background

Nearly half a billion people are dealing with diabetes *mellitus* (DM) [1]. Diabetic retinopathy (DR) affects about one third of DM patients [2], which makes it the leading cause of visual loss in the working-age population [3].

The effects of DM in the retinal vasculature and inner blood–retinal barrier are well established [4] and are the backbone of DR diagnosis and staging. Nonetheless, the retinal blood supply is more complex: the outer retinal layers and the foveal full-thickness retina are supplied by the choroidal vasculature and the associated external blood–retinal barrier [5]. These structures are hardly considered in today’s ocular diabetes practice.

Thanks to enhanced depth imaging (EDI) protocol [6] and swept-source optical coherence tomography (OCT), better in vivo visualization of the choroid is possible. Some groups have focused on choroidal thickness (CT) but it is a coarse measurement for such a complex tissue and no consensus has been reached [7–12]. Moreover, CT is influenced by several local and systemic factors [13–15].

More recently, choroidal vascularity index (CVI) has been described [16, 17]. CVI has been remarkably investigated in DM eyes [18–21]. However, most studies have limitations, namely: considering CVI and CT in non-corresponding and small areas [18–20]; comparing CVI in unstandardized width areas [19]; not stipulating if the suprachoroidal space was considered for analysis [18, 20]; not analyzing absolute choroidal luminal and stromal areas [18, 19] or not considering age and axial length as potential confounders for CVI [18].

Therefore, our purpose was to study the choroid in diabetic patients using CVI and its corresponding stromal, vascular luminal and total choroidal areas, in standardized widths. Secondarily, we aimed to analyze central, nasal, and temporal sectors.

## Patients, materials and methods

### Study design

Cross-sectional observational study set at the Ophthalmology Department of *Centro Hospitalar e Universitário do Porto*, Portugal, during February 2019 and February 2020.

### Study population

DM patients were consecutively recruited, together with age-matched non-diabetic controls from routine appointments. Exclusion criteria were eyes with active macular edema, ischemia, or neovascularization; intraocular procedures in the previous 3 months or signs of concomitant retinal diseases or ocular inflammatory diseases. The estimated sample size (35 subjects per group) was calculated with a previous published formula [22], using the standard deviation of CVI found in previous studies (3%) [19], for a power of 80%, a significance level of 5%, to find a 2% difference in CVI.

Included subjects were further divided in non-DM eyes (group 1), non-DR diabetic eyes (group 2), non-treated DR group (no requirement for treatment, postulating an early stage, group 3) and treated DR group (required treatment with intravitreal anti-VEGF or corticosteroids and/or with focal or panretinal photocoagulation (PRP), as a more advanced stage of the disease, group 4).

### Study protocol

Demographic data and medical history were recorded, namely previous ocular surgeries, photocoagulation with LASER, either focal or PRP, and intravitreal injections of anti-VEGF or corticosteroids. Axial-length was collected from the patients’ charts.

In the study visit, best-corrected visual acuity (BCVA) converted to logMAR, intraocular pressure (IOP) with Goldmann tonometer, slit-lamp biomicroscopy and fundus examination under mydriasis were evaluated. Macular spectral-domain OCT (Heidelberg Spectralis, Germany) high-resolution (1024 horizontal pixels, 20°) imaging was performed with and without EDI protocol [6] and with automatic real-time image averaging set at 100 images. Individual retinal layers were automatically segmented and measured in the central 5 mm, with the proprietary Heidelberg Eye Explorer^®^ software. Each scan was checked for erroneous segmentation.

### Imaging protocol

**Fig. 1:**
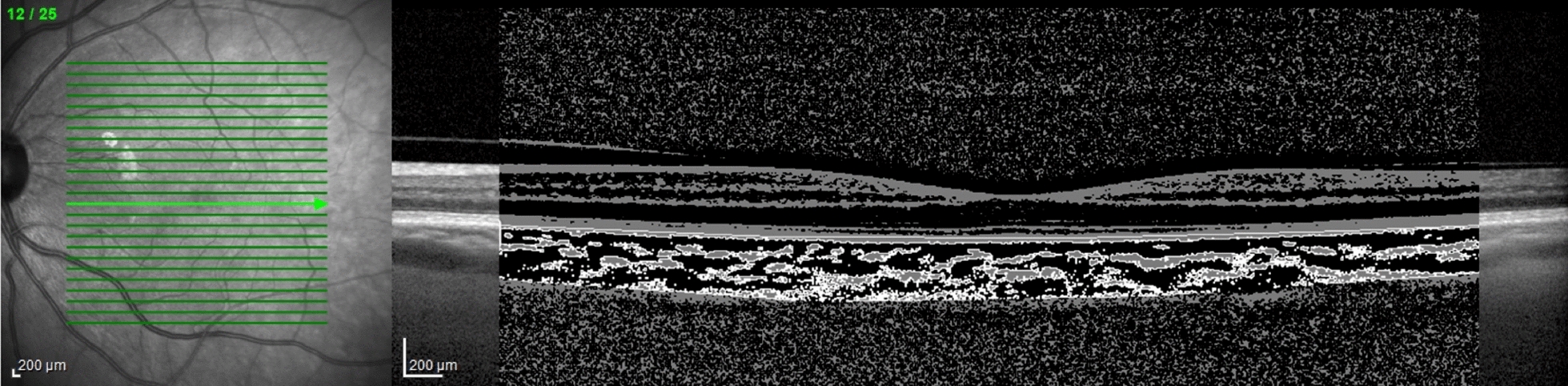
Standardized 5 mm-with binary image over original optical coherence tomography exported image

**Fig. 2:**
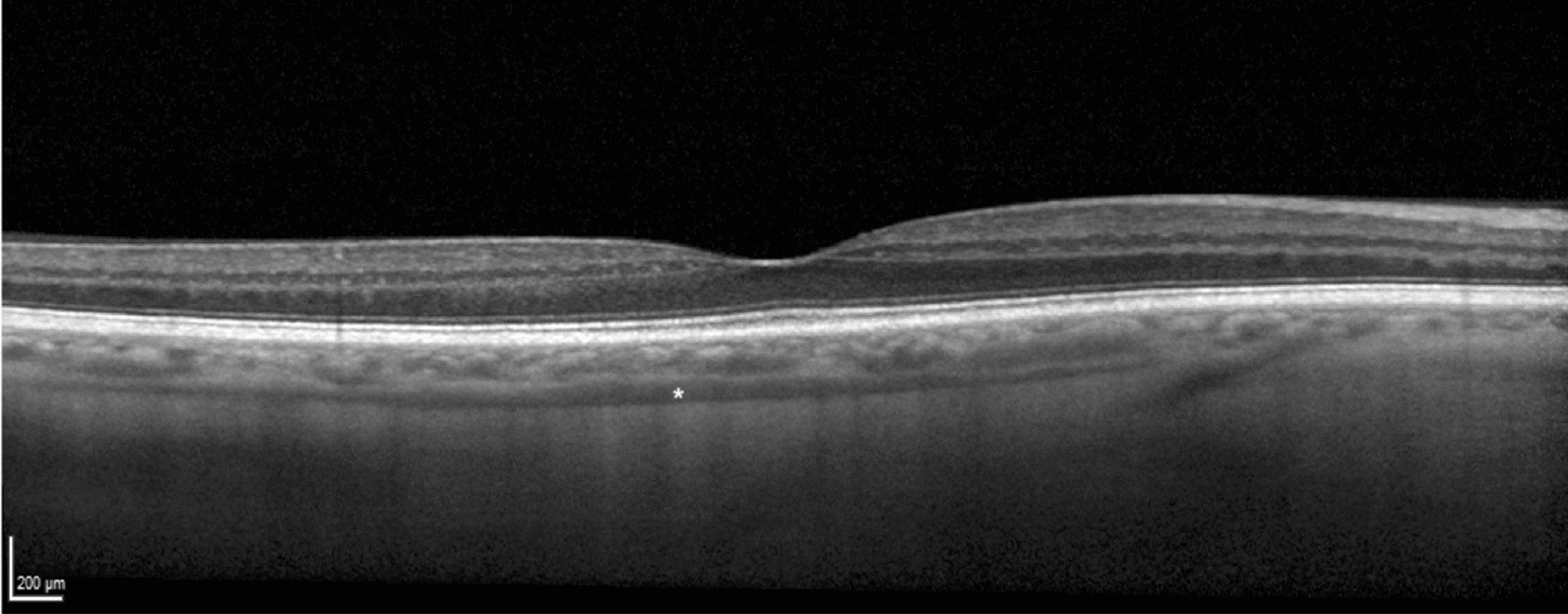
Example of a visible suprachoroidal space, identified with a white asterisk

Scans with ambiguous choroid/sclera boundary, erroneous centration or poor acquisition quality were excluded from analysis. A B-scan centered in the fovea was exported in a 1:1 μm proportion and a semi-automated macro analyzed the choroid (detailed in Additional file 1):

1. The OCT scale was automatically read to calculate its true resolution (μm/pixel) and the image was automatically cropped to a centered 5 mm-width standard, independent of the width in pixels (Fig. 1).
2. Choroid limits were manually drawn one single time by a single investigator who was masked to the patients’ clinical information. This was the only manual step in our protocol. The outer border of the hyperreflective line representing the retinal pigment epithelium was considered the inner choroidal limit. The outer choroidal limit was defined as inner border of the hyperreflective sclera. The presence of a visible suprachoroidal space (SCS), defined as a continuous homogenous hyporeflective layer between the choroid and the sclera (Fig. 2), was recorded and, in such cases, SCS was not considered part of the choroid.
3. Binarization of the OCT image was performed automatically using the same tools as previous studies [16, 17].
4. The choroidal area (CA), hyperreflective stromal area (SA) and hyporeflective vascular luminal areas (LA) were automatically calculated in the full 5 mm-width image (G) and in 1 mm-width central (C), nasal (N) and temporal (T) sectors. CVI was calculated as LA/CA. CA was converted to average CT by dividing it by the corresponding width.

### Statistical analysis

We analyzed only one eye *per* subject, and we always choose the same eye (right eye) because of the slightly asymmetrical arterial supply (due to the aortic anatomy).

After confirmation of normal distribution of variables and homogeneity of variances, Student’s *t-*test was used to compare variables between two groups. One-way ANOVA test was used for multiple group comparison and Fisher’s Least Significant Difference post-hoc analysis was used to adjust the significance. Univariate and multivariate linear regression and Person’s correlation coefficient were used to correlate the outcomes with independent variables. To assess the variability of the outcomes, the coefficient of variation (CV) was calculated as standard deviation divided by mean.

Values are show as mean ± standard deviation, unless otherwise specified. All *p-*values (p) are 2-sided, and p < 0.05 were considered significant.

## Results

### Demographic and clinical data

Initially, 381 subjects were included. Due to ambiguous choroid/sclera boundary, erroneous centration and/or poor acquisition quality, 63 eyes (16.5%) were then excluded. Therefore, we considered for analysis 318 subjects, 58.2% of which were female. They comprised 265 diabetic subjects (67.4 ± 11.9, range 34–90 years-old) and 73 control non-diabetic subjects (72.2 ± 7.5, range 56–85 years-old). The number of subjects, demographic and clinical information *per* group are stated in Table 1.

**Table 1:** Demographic and clinical data compared among groups

	1. Non-DM	2. Non-DR	3. Non-treated DR	4. Treated DR	Total
Subjects (n)	73	39	52	154	318
Age (years)	72.2 ± 7.5	71.3 ± 12.0	66.7 ± 13.0	66.7 ± 11.3	68.5 ± 11.2
Presence of AHT (%)	57	77	77	69	68
Type of DM ½ (n)	–	5/34	4/48	28/126	37/208
Time with DM (years)	–	18.6 ± 13.5	20.9 ± 9.9	25.4 ± 10.9	23.3 ± 11.5
HbA1c (%)	–	7.2 ± 0.9	7.9 ± 1.7	8.1 ± 1.6	7.9 ± 1.6
Time with DR (years)	–	–	5.1 ± 3.0	12.3 ± 8.7	11.1 ± 8.5
BCVA (logMAR)	0.10 ± 0.23	0.16 ± 0.23	0.21 ± 0.22	0.34 ± 0.39	0.25 ± 0.33
Axial length (mm)	23.33 ± 1.03	23.42 ± 1.30	23.36 ± 1.44	22.85 ± 0.78	23.23 ± 1.08

*AHT* Arterial hypertension, *DM* diabetes mellitus, *DR* diabetic retinopathy, *BCVA* best-corrected visual acuity

### Possible confounders

CT-G, SA-G and LA-G diminished with age (p < 0.001). LA-G revealed more pronounced decrease than SA-G (− 0.011 mm^2^/year vs − 0.004 mm^2^/year), so that CVI-G also decreases with age (− 0.09% per year, p < 0.001). The same changes were seen in a subanalysis including only healthy subjects (group 1, p < 0.017 for all).

The prevalence of arterial hypertension (AHT) was higher in DM patients (p = 0.018). It did not affect CVI-G (p = 0.216), even when considering the severity of DR as confounder (p > 0.384 in a subanalysis per group).

Axial length data was available in 127 subjects, and it did not differ among groups (ANOVA, p = 0.148). Contrarily to CT-G (− 20.5 μm *per* mm, p < 0.001), SA-G (− 0.04 mm^2^ *per* mm, p < 0.001) and LA-G (− 0.06 mm^2^ *per* mm, p = 0.001), axial length did not influence CVI-G (p = 0.301) as it is the result of LA and SA that changed in the same direction.

No differences in outcomes were found regarding the type of DM when adjusting for age. No correlation was found between the outcomes and the last HbA1c value.

### Comparison among groups

**Fig. 3:**
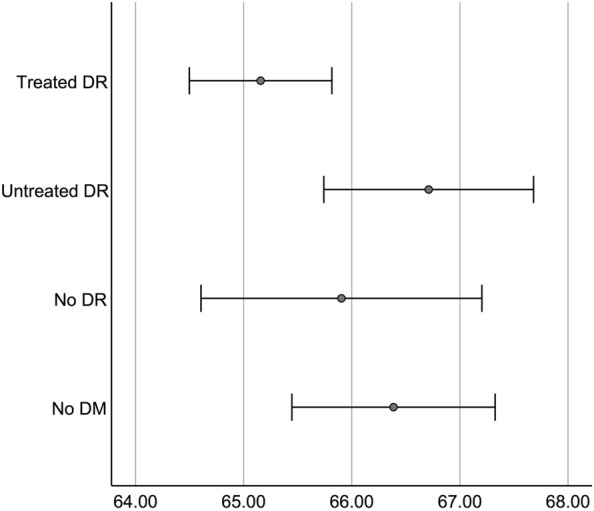
Graph representing choroidal vascularity index by groups. Marks represent mean and error bars represent 95% confidence intervals for mean

A comparison among the multiple groups found a difference in CVI-G and CVI-T (ANOVA test, p = 0.042 and p < 0.001, respectively) but no significant differences in the other outcomes (ANOVA test, p > 0.124). A post-hoc analysis of CVI-G for multiple comparisons is detailed in Table 2 and Fig. 3.

**Table 2:** Post-hoc analysis with Fisher’s Least Significant Difference for multiple comparisons of choroidal thickness in 5 mm-with area

	1. Non-DM	2. Non-DR	3. Non-treated DR	4. Treated DR
Mean ± standard deviation				
CVI-G (%)	66.38 ± 4.03	65.90 ± 4.01	66.7 ± 3.48	65.16 ± 4.14
*p*-value				
1. Non-DM	–	0.544	0.654	**0.031**
2. Non-DR	0.544	–	0.341	0.298
3. Non-treated DR	0.654	0.341	–	**0.016**
4. Treated DR	**0.031**	0.298	**0.016**	–

Statistically significant values are highlighted as bold

*CVI-G* choroidal vascularity index in 5 mm-with area, *DM* diabetes, *DR* diabetic retinopathy

A multivariate analysis adjusting for age confirmed a significant difference in CVI-G between groups 1 and 4 (β = − 0.091%/year, p = 0.001 for age and β = − 1.80%/group, p = 0.013 for the groups) and between groups 3 and 4 (β = − 0.101%/year, p = 0.001 for age and β = − 1.55%/group, p = 0.002 for the groups) but no differences among the other groups (p > 0.403).

### Analysis of the treated-DR group

No differences in outcomes were found according to treatment with insulin (n = 108, p > 0.159 for CVI) or previous treatment with IVI (n = 23, p = 0.238 for CVI). Patients that underwent PRP had a lower CVI (64.08 ± 4.30 vs 66.63 ± 3.43, p < 0.001).

### Associations with visual acuity

**Fig. 4:**
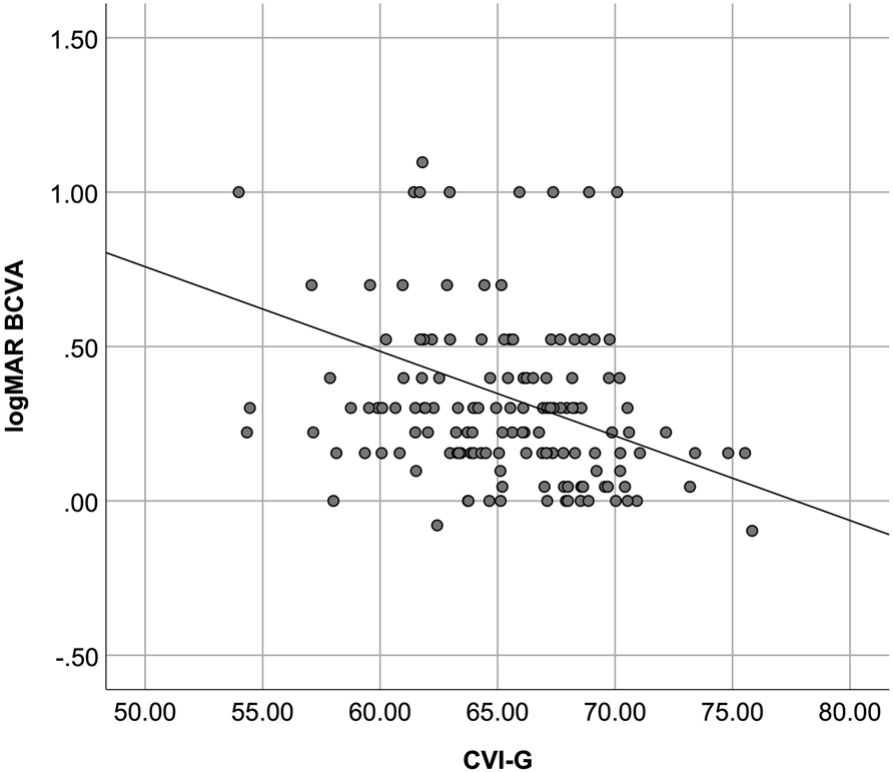
Scatter plot to demonstrate the correlation between choroidal vascularity index and logMAR best-corrected visual acuity (BCVA), in a controlled group of eyes (eyes submitted to panretinal photocoagulation with stable ocular disease and no media opacities)

Table 3 shows possible associations between logMAR BCVA and CT, CVI, full retinal thickness and individual retinal layers thickness, in the full sample and by group, excluding eyes with optical media opacities (n = 214). Figure 4 demonstrates the correlation between CVI and logMAR BCVA. Age, a potential confounding factor, was not associated with logMAR BCVA (p = 0.343).

**Table 3:** Correlations between logMAR best-corrected visual acuity and choroidal and retinal biomarkers, in the full sample and by group, excluding eyes with optical media opacities

	CT-G	CVI-G	RT	NFL	GCL	IPL	INL	OPL	ONL
Full sample									
r	− 0.101	− **0.289**	− 0.138	0.091	− 0.130	− 0.061	− 0.013	0.152	− **0.244**
*p-*value	0.145	**<** **0.001**	0.132	0.322	0.154	0.505	0.885	0.095	**0.007**
No DM									
r	− 0.094	− 0.132	0.080	− 0.148	0.087	0.136	− 0.034	0.234	0.047
*p-*value	0.477	0.315	0.564	0.285	0.532	0.327	0.804	0.088	0.737
No DR									
r	− **0.416**	− 0.079	− 0.299	0.225	− 0.016	− 0.175	− 0.197	− 0.298	− 0.340
*p-*value	**0.025**	0.685	0.177	0.315	0.943	0.435	0.379	0.178	0.122
Non-treated DR									
r	− 0.336	− 0.155	− 0.389	− 0.187	− 0.411	− 0.289	− 0.090	0.139	− 0.006
*p-*value	0.065	0.404	0.073	0.405	0.057	0.192	0.689	0.538	0.979
Treated DR									
r	− 0.079	− **0.362**	− 0.168	− 0.014	− 0.168	− 0.070	0.167	0.099	− 0.390
*p-*value	0.455	**<** **0.001**	0.444	0.951	0.444	0.751	0.446	0.655	0.066

Statistically significant values are highlighted as bold

Average choroidal thickness in 5 mm-width area (CT-G); choroidal vascularity index in 5 mm-with area (CVI-G); full retinal thickness (RT); nerve fibre layer thickness (NFL); ganglion cell layer thickness (GCL); inner plexiform layer thickness (IPL); inner nuclear layer thickness (INL); outer plexiform layer thickness (OPL); outer nuclear layer thickness (ONL) in central 6 mm; Pearson’s correlation coefficient (r)

### Comparison between outcomes

Distribution of outcomes per sector is shown in Table 4. SA-N, LA-N and CT-N were significantly thinner than their T or C counterparts (p < 0.001 for all pairs). CVI-T was lower than its N or C counterparts (p < 0.001).

**Table 4:** Distribution of outcomes per sector

	G (5 mm)	C (1 mm)	N (1 mm)	T (1 mm)
CT (μm)	241.9 ± 78.8	265.1 ± 86.0	236.8 ± 89.5	256.8 ± 80.4
LA (mm^2^)	0.800 ± 0.276	0.176 ± 0.059	0.159 ± 0.063	0.169 ± 0.057
SA (mm^2^)	0.409 ± 0.129	0.089 ± 0.030	0.078 ± 0.029	0.089 ± 0.027
CVI (%)	65.79 ± 4.03	66.08 ± 4.73	66.30 ± 5.18	64.86 ± 5.53

Average choroidal thickness (CT); luminal area (LA); stomal area (SA); choroidal vascularity index (CVI) in the 5 mm-with area (G) and in the central (C), nasal (N) and temporal (T) 1 mm-with areas

CVI provided less intersubject variability than CT (CV 0.061 and 0.326, respectively, in the G sector). Measuring CVI in a 5 mm-width provided less intersubject variability than in central 1 mm-sector C (CV 0.061 and 0.072, respectively).

No choroidal measurement correlated with full thickness retinal thickness or any individual layer thickness (p > 0.177 for all pairs).

### Presence of a visible suprachoroidal space

The SCS was visible in 61 subjects and was associated with a reduced CVI (64.17 vs 66.13%, p < 0.001 for CVI-G). We found no significant differences in our other outcomes regarding a visible SCS (p > 0.185).

## Discussion

To our best knowledge, this is the largest study about the choroid in DM patients and the first one to consider standardized areas (5 mm and 1 mm) and individual sectors (G, C, T and N). Our results corroborate previous reports [18–21] and attempt to overcome some their limitations. For the first time, we relate choroidal structure with visual function.

CVI seems to be a better biomarker of the choroidal vascular structure than CT. It is less influenced by secondary factors than CT, such as axial length and AHT. Nevertheless, CVI decreases with age, even in healthy eyes. Likewise, the retinal vascular density accessed with OCT-angiography decreases with age [23] and, together, these changes may reflect systemic age-related vascular dysfunction [24].

Contrarily, a recent study by the group of Hao Zhou et al., that used swept-source OCT and a fully automated volumetric algorithm, found an inverse correlation of age with vascular volume and with stromal volume but not with CVI. This disparity may be explained by the different age distribution of the groups (55 ± 19 in their sample and 72 ± 7 years in our healthy eyes group). Thus, a non-linear relation between age and CVI may be considered, where CVI decreases with age, later in life.

We report a decrease in CVI in patients with DR that required treatment when compared to normal subjects and to DR patients that did not require treatment, independently of changes in LA, SA, or CT. The fact that our control group was older and that we performed a multivariate analysis reinforces the meaning of our findings regarding a truly reduced CVI due to DR. Despite their limitations, previous studies in DM patients using CVI also found a reduction in CVI as DR worsens [18–21].

Our grouping protocol promotes the differentiation of early stages of the disease and attempts to mimic clinical practice setting. Our methodology excluded patients with active edema, ischemia, or neovascularization. These aggravating factors may be independent causes for changes in choroidal blood flow and for changes in visual function.

Interestingly, we found no significant differences in groups 2 and 3 (early stages) when compared to the control group. The small size of these groups, the different age, the higher variability of measurements or a true absence of difference may justify not reaching significance. Some previous studies have found a reduced CVI in DM patients without DR [25–27], while other have not [18–21]. It remains unclear if diabetic choroidopathy can be detected before classic retinopathy. Considering the different findings and higher variability of CVI in non-DR diabetic eyes, we hypothesize that this group includes eyes in diverse subclinical stages, possibly with different risk of progression to DR. Thus, future studies should analyze prospectively the role of choroidal parameters in predicting the development of DR in diabetic patients.

Globally, our findings relate to choroidal medium and large vascular network attenuation in DR. Additionally, OCT angiography is showing increasing interest in diabetic retinopathy [28]. Most studies [29–31] have reported changes in the choriocapillaris, the small and capillary vessel layer, of diabetic eyes even with no clinical signs of retinopathy [31]. In addition, in healthy eyes, choriocapillaris flow in OCT-A seems to have a relation with choroidal stroma and vascular lumen in structural OCT [26]. A multimodal approach will probably achieve more elucidative conclusions.

Regarding associations with visual function, in the full sample, the outer nuclear layer thickness correlated with BVCA. This association has been previously described in diabetic patients [32]. However, in the present study, the correlation of BCVA with CVI was stronger and independent of the outer nuclear layer thickness. In the treated-DR subgroup, BCVA correlated only with CVI. The correlations in the full sample must be interpreted carefully as they may be driven by the treated DR group. It is unclear if this is a cause or a consequence of a common process: the retinal photoreceptors depend on the choroid [5] for their nutrient supply but, on the other side, the choroid may suffer changes after photoreceptor atrophy [33]. Choroid vascular status may help understand the different visual acuity sometimes observed in DR, that retinal biomarkers cannot fully explain.

We found the 5 mm-width area to be more sensitive than 1 mm-width area and, additionally, to have lower variability. Agrawal et al. compared, in normal eyes, three different scanning areas: single full-width foveal scan; volumetric central 1 mm scans and volumetric total macular scans. They reported good reliability using single full-width foveal scans [34]. Additionally, we excluded the SCS as part of the choroid. Otherwise, it might have influenced the results, as it would have been considered vascular lumen and modify average CVI.

In our study, using SD-OCT with EDI protocol, we excluded 16.5% of images due to ambiguous choroid/sclera boundary, erroneous centration and/or poor acquisition quality. Theoretically, the choroid/sclera outer limit visualization worsens as CT and SA (where most choroidal pigment lays) increase. The use of swept-source OCT, with increased penetration through melanin, might overcome this limitation. Our study has some other limitations: its cross-sectional design; the manual and single time drawing of choroidal limits (although it was done by a single investigator to potentiate precision) and the uneven number of eyes in each group (which is a reflex of our methods and of real-life clinical practice).

Choroid investigation should continue, and it may contribute to improved staging of diabetic retinopathy. CVI may be a good biomarker of retinal and visual function in treated diabetic patients. Subsequent prospective studies should focus on how the choroid and CVI may predict the risk of developing DR in DM patients.

## Supplementary Information

**Additional file 1.** Detailed imaging protocol.

## Data Availability

Raw data is available on request to the corresponding author (JHM).
