# Supplementary material for: Choroidal changes and associations with visual acuity in diabetic patients
Source: Int J Retina Vitreous. 2022 Jan 8;8:6. doi: 10.1186/s40942-021-00355-z (PMC8742927; doi:10.1186/s40942-021-00355-z)
Supplement: Supplementary file 1 — Additional file 1. Detailed imaging protocol. [file 40942_2021_355_MOESM1_ESM.pdf]

Algorithm to automatically calculate choroidal parameters

1. A foveal horizontal high-resolution (1024 pixel-width) B-scan was exported from Heidelberg Eye Explorer® software in TIFF format and 1:1 µm proportion.

2. Image import in ImageJ® software. The scale was automatically read to calculate its true resolution (µm/pixel).

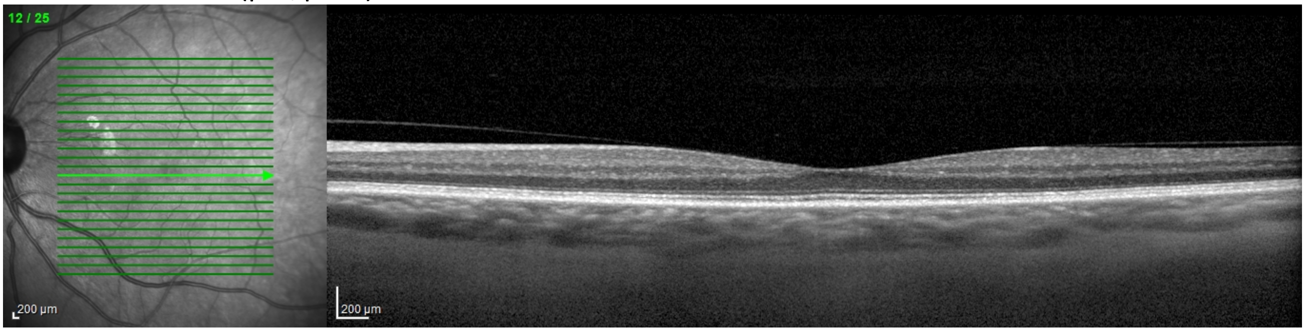

3. The image was automatically cropped to a centered 5mm-width standard, independent of the width in pixels.

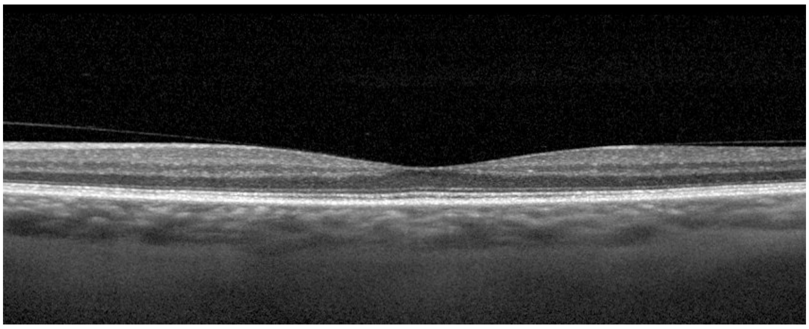

4. The choroid limits were manually drawn using "Polygon selection" by a single investigator. The outer border of the hyperreflective line representing the retinal pigment epithelium was considered the inner choroidal limit. The outer choroidal limit was defined as inner border of the hyperreflective sclera. The presence of a visible suprachoroidal space (SCS), defined as a continuous homogenous hyporeflective layer between the choroid and the sclera, was recorded and, in such cases, SCS was not considered part of the choroid.

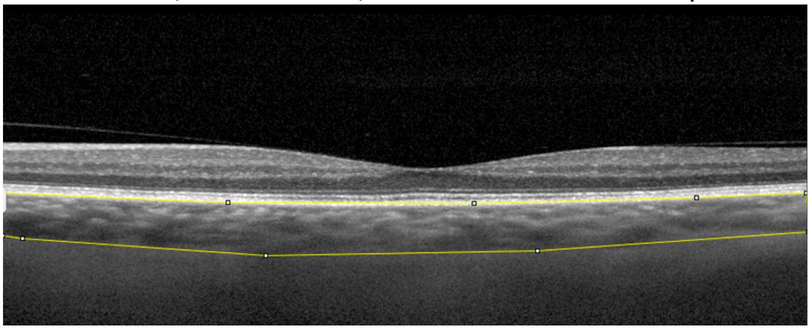

5. Image binarization using "Auto Local Threshold"; “method=Niblack” and “radius=15”.

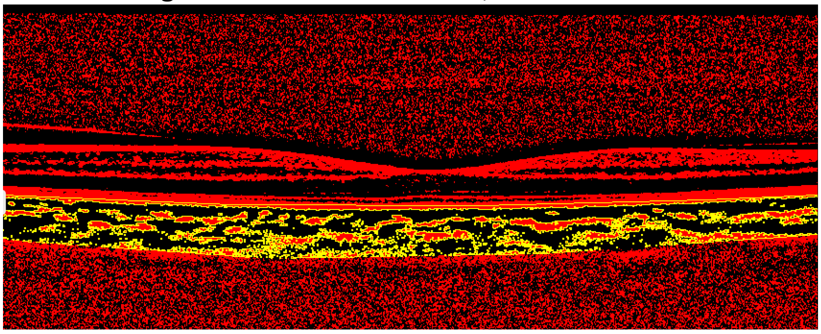

6. The choroidal area (CA), hyperreflective stromal area (SA) and hyporeflective vascular luminal areas (LA) were automatically calculated in the full 5mm-width image (G) and also in 1mm-width central (C), nasal (N) and temporal (T) sectors. CVI was calculated as LA/CA. CA was converted to average CT in the corresponding areas (G, C, N and T).

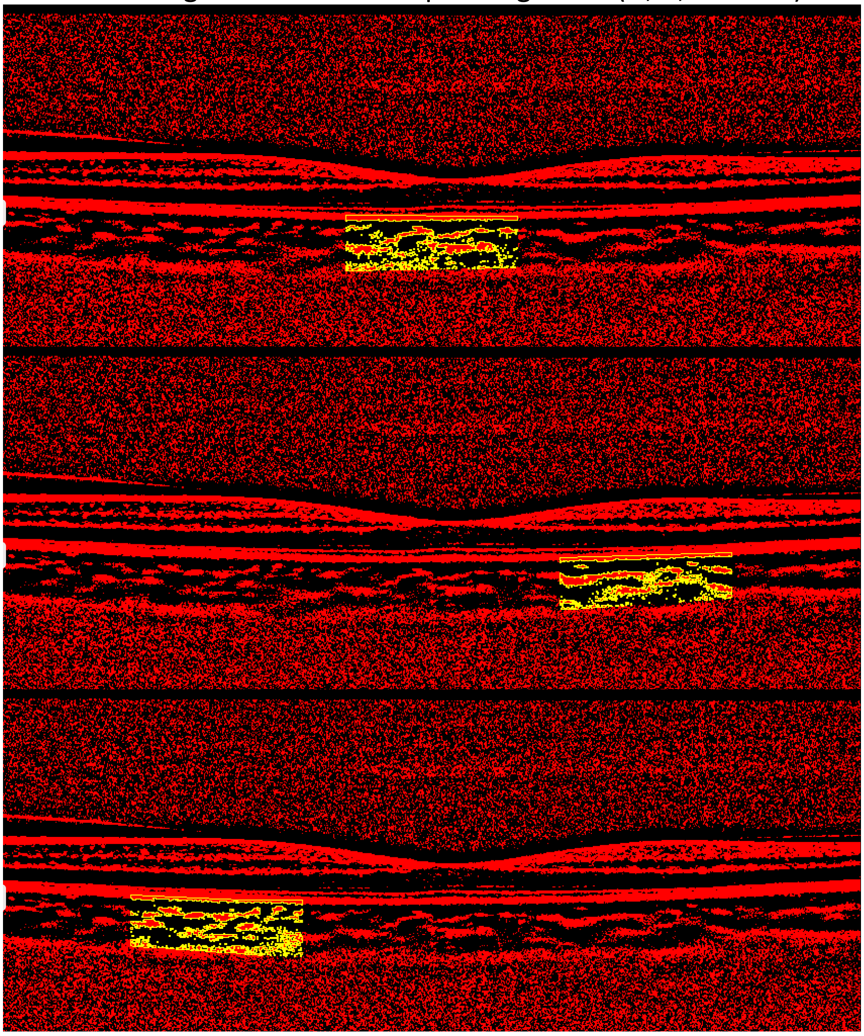

Full macro for use in ImageJ 1.52v:

```
waitForUser("Load Image")
imagename=getTitle()
largura=getWidth
largurafinal=largura-1024
altura=getHeight
makeRectangle(largurafinal, 0, 1024, altura);
run("Crop");
run("Duplicate...","title=11")
makeRectangle(10, 240, 2, 150);
run("Crop");
run("8-bit");
setThreshold(220, 255);
run("Create Selection");
roiManager("Add");
roiManager("Select", 0);
roiManager("Measure");


selectWindow(imagename)
cortefinal=(getResult("Area",0)*(12.5))
sobras=(d2s(((1024-cortefinal)/2),0))
altura=getHeight
makeRectangle(sobras, 0, cortefinal, altura);
run("Crop");
makeRectangle(((cortefinal/2)-(cortefinal/10)), 0, (cortefinal/5), altura);
roiManager("Add");
makeRectangle(((cortefinal/4)-(cortefinal/10)), 0, (cortefinal/5), altura);
roiManager("Add");
makeRectangle(((cortefinal*(3/4))-(cortefinal/10)), 0, (cortefinal/5), altura);
roiManager("Add");
run("Select None");
selectWindow(imagename)
setTool("polygon");
waitForUser("Draw choroidal limits")

roiManager("Add");
selectWindow(imagename)
run("8-bit");
run("Auto Local Threshold...", "method=Niblack radius=15 parameter_1=0 parameter_2=0
white");
setThreshold(250, 255);
run("Create Selection");
waitForUser("CT")

roiManager("Add");
```

```
roiManager("Select", newArray(1,4));
roiManager("AND");
roiManager("Add");
roiManager("Select", newArray(2,4));
roiManager("AND");
roiManager("Add");
roiManager("Select", newArray(3,4));
roiManager("AND");
roiManager("Add");
roiManager("Select", newArray(4,5));
roiManager("AND");
roiManager("Add");
roiManager("Select", newArray(5,6));
roiManager("AND");
roiManager("Add");
roiManager("Select", newArray(5,7));
roiManager("AND");
roiManager("Add");
roiManager("Select", newArray(5,8))
roiManager("AND");
roiManager("Add");
roiManager("Select", newArray(4,6,7,8,9,10,11,12));
roiManager("Measure");

waitForUser("Limpar")
roiManager("Select", newArray(0,1,2,3,4,5,6,7,8,9,10,11,12));
roiManager("Delete");
run("Clear Results");
selectWindow(imagename)
close()
selectWindow(11)
close()
```
